# Privacy-Preserving Federated Deep Learning for Robust Anomaly Detection in Distributed Security Sensing Systems

**DOI:** 10.3390/s26123901

**Published:** 2026-06-19

**Authors:** Di Xu, Hongli Chen, Yansen Zeng, Yifan Yang, Jinghan Huang, Jiarui Song, Yan Zhan

**Affiliations:** 1 China Agricultural University, Beijing 100083, China; 2 National School of Development, Peking University, Beijing 100871, China; 3 China School of Banking and Finance, University of International Business and Economics, Beijing 100029, China

**Keywords:** artificial intelligence-driven sensing, distributed security sensing, edge intelligence, non-IID data, intelligent IoT security

## Abstract

With the widespread adoption of intelligent terminals, edge devices, and distributed information systems in the financial domain, financial security sensing data exhibit multisource heterogeneity, dynamic temporal patterns, and high privacy sensitivity. Traditional centralized anomaly detection methods are no longer able to simultaneously satisfy the requirements of cross-institutional or cross-node collaborative modeling, client data privacy protection, and robust monitoring of transaction and system anomalies. To address this challenge, a data-local federated deep anomaly detection framework has been proposed for distributed financial security sensing systems. Initially, a local deep financial security sensing representation module is constructed to perform temporal encoding and attention-based modeling on multisource financial signals, including terminal operation status, network transaction communication, backend server operation, identity authentication, and anomaly alerts, thereby extracting representations relevant to anomalous behaviors. Subsequently, a data-local federated optimization and personalized aggregation mechanism is developed to enable cross-node knowledge sharing without transmitting raw transaction or client data, while local personalized detection heads are employed to adapt to non-independent and identically distributed (non-IID) financial institution data. Furthermore, an adversarially robust security detection and trust-aware aggregation strategy is introduced to enhance model stability under input noise, feature masking, anomaly camouflage, and potential malicious client updates. Experimental results demonstrate that the proposed method achieves an Accuracy of 92.37%, a Precision of 89.41%, a Recall of 88.26%, an F1-score of 88.83%, an AUC of 93.06%, and a PR-AUC of 89.15% in the primary financial anomaly detection task, significantly outperforming baseline methods such as Isolation Forest, Autoencoder, LSTM, Transformer, FedAvg, FedProx, SCAFFOLD, and MOON. In robustness experiments, the method attains F1-scores of 87.95%, 86.42%, 86.88%, 84.57%, 86.73%, and 83.91% under Gaussian noise, feature masking, temporal shift, adversarial perturbation, and 20% and 30% malicious client scenarios, respectively. Ablation studies further confirm the effectiveness of local representation learning, personalized federated optimization, adversarial training, and trust-aware aggregation mechanisms. Overall, the proposed approach provides an efficient intelligent anomaly detection solution for financial AI security monitoring scenarios characterized by data localization requirements, node heterogeneity, and attack perturbations.

## 1. Introduction

With the rapid development of the Internet of Things, edge computing, intelligent terminals, and distributed information systems in financial and fintech scenarios, large volumes of security-related data are continuously generated by network infrastructures, edge nodes, sensing devices, and financial business systems [1]. Compared with conventional single-source security logs, modern distributed financial security sensing data exhibit multisource heterogeneity, dynamic temporal characteristics, and high-dimensional complexity, encompassing transaction network traffic, access logs, system alarms, terminal operating states, service request behaviors, resource-load indicators, client interaction trajectories, and anomalous event records [2]. Furthermore, from the perspective of IoT security awareness and edge intelligence, distributed financial sensing data are evolving toward increasingly complex dimensions. Current sensing systems are no longer restricted to one-dimensional system logs or network traffic features, but are rapidly expanding into multimodal and multimedia dimensions, such as financial video surveillance and transactional audio analysis. For instance, recent studies have highlighted the critical importance of multimedia data security in edge financial environments, exploring sophisticated video cryptosystems and chaotic encryption techniques [3]. Modeling such complex data using artificial intelligence has become a key direction in intelligent financial security sensing, AI-driven risk monitoring, and edge-intelligent financial security [4]. In financial transaction systems, digital banking, smart financial cities, and intelligent payment networks, anomalous behaviors are often stealthy, sudden, and capable of cross-node propagation; delayed detection may result in client information leakage, transaction interruptions, or system-level security incidents [5]. Therefore, intelligent anomaly detection methods capable of adapting to distributed financial environments while ensuring privacy protection and robustness possess significant theoretical and practical value [6].

Traditional security anomaly detection methods primarily rely on rule matching, statistical analysis, and shallow machine learning models [7]. Threshold-based methods detect attacks through manually defined conditions, statistical methods identify abnormal transaction flows via distributional deviations, and support vector machines, random forests, or clustering techniques classify anomalies based on handcrafted features [8]. Although these methods have shown effectiveness in early financial security monitoring tasks, their limitations have become increasingly apparent with the expansion of distributed financial systems and the growing complexity of attacks [9]. First, manually designed features are insufficient for automatically extracting deep anomaly patterns from high-dimensional, multisource, and dynamically evolving financial time-series data [10]. Second, centralized detection frameworks require data from multiple nodes to be uploaded to a central server, which incurs communication and storage overhead and may expose sensitive transaction information, client behavior trajectories, and system-state data [11]. In addition, real financial distributed environments are characterized by substantial data heterogeneity, where behavioral patterns and anomaly types vary across devices, systems, and time periods, resulting in limited cross-node generalization for centralized models [12]. Security sensing tasks are also inherently adversarial, as attackers may forge input signals, inject anomalous noise, or contaminate training data, rendering traditional methods insufficiently robust [13].

Recently, deep learning has been widely applied to enhance financial security anomaly detection performance [14]. CNNs can capture local anomalous fluctuations within temporal windows. RNNs, LSTMs, and GRUs can model dynamic sequential dependencies, and Transformer architectures with self-attention mechanisms can capture long-range temporal correlations and multivariate coupling relationships [15]. Autoencoders, graph neural networks, and contrastive learning approaches have also been employed for anomaly detection, attack classification, and transactional behavior analysis [16]. Compared with conventional machine learning, deep learning enables automatic learning of nonlinear feature representations, thereby providing superior capability for analyzing high-dimensional financial temporal data [17]. However, most existing deep learning-based financial security detection methods remain dependent on centralized training, which conflicts with privacy protection requirements [18]. Moreover, conventional deep models typically assume independent and identically distributed data, whereas distributed financial nodes often exhibit strong non-IID characteristics in anomaly ratios, business loads, device states, and attack patterns, causing client drift and global model degradation in traditional federated learning [19]. Existing federated learning methods remain vulnerable to evasion attacks and malicious client updates, which may perturb input data or contaminate global optimization through abnormal gradients [20]. Therefore, privacy-preserving cross-node anomaly detection with stable performance under non-IID distributions and adversarial attacks remains a critical challenge [21]. Ali Alferaidi et al. [22] integrated BERT with deep neural networks for healthcare IoT intrusion detection. Swapna et al. [23] combined GAN, blockchain, and differential privacy to enhance federated learning robustness against poisoning and privacy attacks. Suvathika et al. [24] proposed BR-DP-FedDF by integrating Byzantine-robust aggregation and differential privacy for IoT big data. Mahor et al. [25] combined deep learning with secure authorization for privacy-preserving child behavior monitoring. Shofiullah et al. [26] developed a federated learning framework for distributed manufacturing quality prediction and supply chain optimization.

To address these challenges, a privacy-preserving federated deep learning anomaly detection framework is proposed for distributed financial security sensing systems. Cross-node collaborative modeling is achieved without sharing raw data, while deep temporal feature extraction, personalized federated optimization, and adversarially robust training are integrated to enhance anomaly detection in complex financial security environments. Specifically, a local deep security sensing representation module is constructed to model multidimensional security signals using Transformer architectures, temporal encoders, and attention mechanisms, enabling anomaly-related representations to be learned from system states, access behaviors, network traffic, and service requests. A personalized federated collaborative optimization mechanism is then designed for non-IID scenarios, wherein a shared encoder enables cross-node knowledge sharing and local detection heads preserve client-specific characteristics, mitigating model drift caused by heterogeneous data. Finally, an adversarially robust security detection and trust-aware aggregation mechanism is incorporated, in which adversarial training strengthens resistance to input perturbations and anomaly camouflage, while client trust evaluation and robust aggregation reduce the influence of malicious updates on the global model.

The main contributions of this study are summarized as follows:

1. The primary contribution lies in the end-to-end integration of a privacy-preserving federated deep learning framework, which synergistically addresses multidimensional feature extraction, non-IID client drift, and adversarial vulnerabilities in distributed financial security sensing systems without sharing raw data.
2. A local deep security sensing representation module is designed as a core component to extract anomaly-related features from multidimensional state sequences, access behaviors, and system operation indicators through temporal deep encoding and attention mechanisms.
3. A personalized federated optimization mechanism is constructed to address non-IID data distributions, explicitly separating a shared encoder for cross-node global knowledge sharing from local detection heads for client-specific adaptation, thereby mitigating model drift.
4. A dual-defense adversarially robust security detection and trust-aware aggregation strategy is embedded into the framework to enhance stability against client-side input perturbations and server-side poisoning attacks.
5. Extensive evaluations in a multi-node distributed experimental environment based on financial transaction security and system operation state data demonstrate that the synergistic integration of these components significantly outperforms existing baselines in terms of anomaly detection, non-IID generalization, adversarial robustness, and malicious client defense.

## 2. Related Work

### 2.1. Deep Learning for Financial Security Sensing and Anomaly Detection

With the rapid development of intelligent Internet of Things, edge computing, and distributed sensing systems in financial technology, digital banking, and online payment scenarios, the scale and complexity of financial security sensing data have increased substantially, rendering rule-based and shallow machine learning methods increasingly insufficient for anomaly identification in complex transaction environments, cross-node business systems, and dynamic attack scenarios [27]. Deep learning has therefore been widely applied to anomaly detection in cybersecurity, financial risk control, industrial monitoring, and intelligent sensing systems because of its nonlinear modeling and automatic representation learning capabilities [28]. By hierarchically abstracting high-dimensional inputs through multilayer neural networks, latent patterns, risk signals, and anomalous features embedded in complex financial data can be automatically learned [29]. Compared with handcrafted-feature-based methods, deep models can extract temporal correlations, behavioral dependencies, and multivariate coupling relationships from raw security sensing data through end-to-end training, demonstrating stronger adaptability in distributed financial security scenarios [30].

In financial security sensing tasks, convolutional neural networks can capture local fluctuations within temporal windows and identify anomalous patterns in network traffic, system loads, and transactional access behaviors [31]. Recurrent neural networks and long short-term memory networks model temporal dependencies through hidden-state propagation and are suitable for modeling financial transaction behaviors, device states, and access sequences [32]. Gated recurrent units reduce model complexity while preserving temporal modeling efficiency [33]. Transformer and self-attention mechanisms further enable global correlations among temporal segments and heterogeneous features to be captured, allowing anomaly-related regions associated with transactions, access, and system risk to be emphasized more effectively [34]. Autoencoders detect anomalies via reconstruction errors, whereas graph neural networks learn attack propagation or risk diffusion patterns from account nodes, transaction relationships, and network structures [35]. Recent studies have further extended the application of anomaly detection models to highly complex digital risk environments, such as blockchain and modern financial ecosystems. For example, in distributed and structurally complex financial data scenarios, network-based anomaly detection in blockchain transactions has been actively explored using graph neural networks and density-based spatial clustering with noise [36]. Similarly, in the domain of financial transaction security, behavioral and transactional feature engineering combined with machine learning approaches has demonstrated significant potential for fraud detection in banking systems [37]. These advancements closely align with experimental focuses on financial transaction security and system operational states, highlighting the necessity of handling multisource behavioral indicators and structurally complex financial data representations. These methods have achieved promising results in intrusion detection, industrial anomaly sensing, financial risk identification, and intelligent device monitoring [38].

However, most existing deep security detection methods remain dependent on centralized training, where data from different devices, financial institutions, or edge nodes are uploaded to a central server [39]. Although this paradigm can improve model performance, it is unsuitable for many real distributed financial security sensing scenarios [40]. Sensitive records, including transaction logs, account access trajectories, operational system states, and terminal device behaviors, may be exposed if uploaded directly, and such centralized processing is difficult to reconcile with strict data compliance requirements. Moreover, centralized training usually assumes similar training and testing distributions, whereas real multi-node financial environments often exhibit significant heterogeneity [41]. Variations in business types, client behaviors, anomaly ratios, access frequencies, and attack patterns make it challenging for a single centralized model to balance global generalization and local adaptation. Since financial security sensing data and attack patterns may also evolve over market conditions, business cycles, and attack strategies, achieving cross-node collaborative security modeling without aggregating raw data has become a key problem in distributed financial security sensing [42,43].

### 2.2. Federated Learning for Privacy-Preserving Financial Edge Intelligence

To resolve the conflict between financial privacy protection and cross-node collaborative learning, federated learning has emerged as an important paradigm in edge intelligence and privacy-preserving computing [44]. In federated learning, model training is performed locally on each client, and only model parameters or gradient updates are uploaded to a central server for aggregation [45]. Since raw transaction, client behavior, and system operational data are not directly shared, the risk of data leakage is reduced compared with centralized learning [46]. FedAvg performs global optimization via weighted averaging of client parameters and remains a classical method for collaborative model construction across distributed nodes [47]. Improved methods, including FedProx, SCAFFOLD, FedNova, and MOON, have been proposed to mitigate data heterogeneity, client drift, and communication efficiency issues [48]. Specifically, FedProx constrains local deviation from the global model, SCAFFOLD corrects gradient drift using control variates, FedNova normalizes heterogeneous local training steps, and MOON enhances global consistency via model contrastive learning [49]. These methods have demonstrated effectiveness in edge intelligence, mobile computing, and distributed learning [50].

Nevertheless, existing federated learning methods remain limited in distributed financial security sensing tasks [51]. Financial security anomalies are often scarce and imbalanced, and different clients may contain different business scenarios, transaction types, or attack categories, or may even lack certain anomalies, making global aggregation insufficient for learning stable anomaly representations [52]. Additionally, real financial security sensing data often exhibit temporal dependencies and dynamic changes, whereas standard federated learning methods are more commonly designed for static classification and may be inadequate for complex transaction sequences, system states, and access behavior sequences [53]. Federated learning systems themselves also face security risks [54]. Since the server cannot inspect client raw data, malicious financial nodes or compromised clients may upload abnormal gradients, inverted parameters, or backdoor information, thereby disrupting global model convergence [55]. Such poisoning and malicious update attacks are particularly critical in financial security sensing, as attackers may deliberately degrade fraud detection, anomalous transaction identification, and system intrusion monitoring by contaminating federated training [56]. Therefore, privacy protection, heterogeneous data adaptation, and malicious attack defense must be jointly considered within federated learning frameworks [57].

### 2.3. Robust and Adversarial Learning for Secure Distributed Financial Sensing

To enhance model robustness in financial security environments, adversarial learning and robust training have increasingly been incorporated into security detection and federated learning [58]. Adversarial learning generates perturbed samples during training so that models encounter attack-like disturbances in advance and become more stable under anomaly camouflage, input noise, and transactional perturbations during testing [59]. Typical methods compute input gradients and add small perturbations to the original inputs, encouraging models to learn stable rather than fragile features [60]. In financial security sensing tasks, adversarial training can strengthen detection capability under evasion attacks, anomalous camouflage, and data perturbations [61]. For instance, attackers may hide anomalous behaviors by modifying transaction frequencies, adjusting access behaviors, altering payment paths, or perturbing partial traffic fields to bypass detection models [62]. Through adversarial training, more robust anomaly representations can be learned, improving detection stability in complex financial attack environments [63].

Robust aggregation methods are also used to protect federated learning systems from malicious clients [64]. Traditional FedAvg assumes that all client updates are trustworthy, whereas some clients in real distributed financial environments may be compromised or deliberately upload abnormal parameters [65]. To address this issue, Median Aggregation, Trimmed Mean, Krum, and Robust Federated Aggregation have been developed to filter abnormal updates, reduce the weights of suspicious gradients, or select clients with consistent update directions [66]. Median aggregation suppresses extreme values through statistical medians, Trimmed Mean removes abnormal gradient components, and Krum selects reliable updates according to parameter distances [67]. Although these methods mitigate model poisoning to some extent, most studies focus on standard tasks such as image classification, and their adaptability to temporally heterogeneous distributed financial security sensing data remains limited [1].

Overall, despite substantial progress in deep anomaly detection, federated learning, and robust optimization, a unified framework for distributed financial security sensing remains lacking [68]. Existing deep security detection methods have difficulty simultaneously supporting financial data privacy protection and cross-node collaboration, whereas conventional federated learning is unstable under non-IID financial data distributions and malicious client attacks. Moreover, most robust aggregation methods are not specifically designed for complex temporal financial security sensing data [69]. Therefore, local deep financial security representation learning, personalized federated optimization, and adversarially robust aggregation should be jointly designed to satisfy privacy protection, heterogeneous data adaptation, and robust security detection requirements [70]. The proposed approach is developed under this motivation by integrating deep temporal modeling, personalized federated learning, and trust-aware robust aggregation for privacy-preserving anomaly detection in complex distributed financial security environments.

## 3. Materials and Method

### 3.1. Data Collection

A hybrid dataset comprising real financial operational records and simulated security sensing signals was constructed using a distributed financial security sensing system as the experimental scenario. The data collection period spanned from January 2023 to December 2024, with continuous collection covering regular working days, holidays, month-end clearing and settlement periods, mid-year and year-end transaction peaks, nighttime system maintenance windows, and periods of concentrated security alarms, as summarized in Table 1. To simulate multi-node deployment, local data storage, and collaborative model training in real distributed financial systems, 10 distinct federated clients were constructed. These clients correspond to core transaction servers, third-party payment gateway servers, front-end business terminals, self-service financial terminals, edge access gateways, core switches, firewalls, identity authentication servers, database servers, and computer-room monitoring devices. Each client retained only locally collected raw data. Prior to modeling, sensitive fields such as client identifiers, transaction serial numbers, terminal identifiers, device addresses, and node names were hashed or irreversibly anonymized. Ultimately, only time-window-level statistical features, device operating-state features, access behavior features, and anomaly-state labels were retained.

Service request sensing data were collected from core transaction servers and interface access gateways. Server-side log probes and interface monitoring agents were used to synchronously record features within 1-min time windows, including the number of requests per unit time, task submission frequency, request failure rate, repeated request ratio, number of abnormal return codes, and request response status. These features were used to characterize access intensity fluctuations and anomalous request patterns in financial service links. Link response data were collected from third-party payment gateways, task scheduling services, and transaction status callback services. Gateway traffic sensors and interface status monitoring modules recorded features such as request response latency, task confirmation time, timeout return counts, repeated callback counts, and abnormal state transitions. Terminal status data were collected from front-end business terminals, self-service financial terminals, and mobile access terminals using built-in terminal status collectors to record device online status, connection interruptions, terminal restarts, short-term high-frequency requests, and abnormal operation counts. Network communication data were collected from edge access gateways, core routers, switch mirror ports, and hardware firewalls using a passive traffic collection method. Only inbound and outbound traffic, connection counts, packet rates, port access frequencies, abnormal connection ratios, and short-connection bursts were recorded, while raw service packet contents were not parsed. Server operation status data were collected from service application servers, database servers, and computing nodes. Server hardware monitoring sensors and host performance probes collected CPU temperature, CPU utilization, memory usage, disk read/write rates, network interface throughput, power status, and fan speed at 30 s intervals, which were further aggregated into mean, maximum, standard deviation, and rate of change over 1-min or 5-min windows. Identity authentication and terminal access behavior data were collected from unified identity authentication servers, login audit systems, and office access terminals, including login frequency, login failure counts, session duration, device switching counts, abnormal access flags, and interface invocation frequency. Computer-room environmental data were collected from server cabinets and environmental monitoring devices, including temperature, humidity, power fluctuation, and local cooling status.

Anomaly event labels were initially collected from intrusion detection devices, firewall alarm systems, risk-rule engines, and security audit platforms. To ensure ground truth reliability, a rigorous label generation and manual verification procedure was implemented. Automated security systems first generated preliminary anomaly flags, which were subsequently reviewed by a panel of cybersecurity and financial risk experts. False positives were filtered, and confirmed events were annotated, including transaction bursts, forged requests, abnormal logins, service latency anomalies, system load anomalies, and abnormal terminal restarts. To supplement rare attack vectors, simulated stealthy attack records were carefully injected into local data pools, forming a comprehensive and robust hybrid dataset encompassing financial transaction security and system operational states.

Since the proposed framework is designed for non-independent and identically distributed federated learning, the heterogeneity across participating nodes was analyzed. As detailed in Table 2, the data distribution across the 10 federated clients exhibits significant variations in total sample size, anomaly ratio, primary anomaly classes, and feature characteristics. This highly skewed distribution confirms that the experimental environment realistically reflects the distributed heterogeneity of actual financial security sensing networks rather than relying on strictly controlled or uniform assumptions.

### 3.2. Data Augmentation

In distributed security sensing systems, raw data collected from heterogeneous nodes are characterized by complex sources, high dimensionality, and inconsistent sampling frequencies. Directly feeding raw data into deep models degrades anomaly detection performance. Therefore, raw data must be uniformly preprocessed and augmented to convert security sensing signals into standardized temporal representations. We achieve temporal alignment by statistically aggregating discrete events within fixed time windows Δt:

(1) $$X_{t}=\frac{1}{N_{t}}\sum_{i=1}^{N_{t}}x_{i},$$

where $N_{t}$ denotes the number of events, and $X_{t}$ denotes the aggregated feature vector. Continuous features are subsequently normalized to eliminate scale differences using standardization or min-max normalization. To address missing values caused by network anomalies, we apply forward filling or mean imputation depending on the temporal continuity of the sensing signals.

A critical challenge in distributed security sensing is the effective representation of behavioral risk factors. For high-dimensional discrete behavioral variables, such as user interaction trajectories, request patterns, and access sources, traditional one-hot encoding leads to excessive dimensionality. Therefore, we utilize an embedding representation to map these discrete behavioral categories into a low-dimensional continuous vector space:

(2) $$e_{i}=W_{emb}c_{i},$$

where $c_{i}$ denotes the original categorical behavioral variable, $W_{emb}$ denotes the embedding matrix, and $e_{i}$ denotes the corresponding continuous embedding vector. This embedding process enables the model to capture latent semantic relationships among distinct user behaviors across different clients. Drawing upon recent methodologies in digital behavioral modeling [71], we leverage these learned embeddings not merely for feature compression, but to actively identify and interpret influential risk factors. By analyzing the learned weights and activation patterns associated with these embedded behavioral indicators post-classification, the framework can effectively differentiate normal operational habits from anomalous access trajectories, thereby providing an interpretable basis for risk-related predictions.

Furthermore, to capture the inherent periodicity of security sensing data, temporal features are periodically mapped using sine and cosine encodings. A sliding-window strategy is then adopted to generate temporal training samples with a fixed window length *T*:

(3) $$X_{t}=\left\{x_{t-T+1},x_{t-T+2},\cdots ,x_{t}\right\}.$$

To improve model robustness in adversarial environments without altering original data semantics, we introduce multiple data augmentation strategies. We simulate sensor errors and log missingness by applying Gaussian noise $x^{\prime }=x+\epsilon$ and random masking via a Bernoulli distribution $x^{\prime }=m⊙x$. We also employ temporal cropping and shifting to adapt to anomalous behaviors occurring at varying temporal scales. To enhance robustness against evasion attacks, a lightweight adversarial sample generation mechanism is introduced using the fast gradient sign method:

(4) $$x_{adv}=x+\epsilon \cdot sign\left(\nabla_{x}L\left(x,y\right)\right),$$

where L(x,y) denotes the model loss function, and ϵ denotes the perturbation strength. Finally, we artificially construct polluted samples with reversed updates and random gradients to simulate realistic federated poisoning attacks, enabling the model to adapt to malicious update behaviors in advance and improving the effectiveness of subsequent trust-aware aggregation strategies.

### 3.3. Threat Model

To comprehensively evaluate the robustness of the proposed privacy-preserving federated anomaly detection framework, it is essential to clearly define the security threat model and the assumed capabilities of potential attackers. In our distributed security sensing scenario, we assume the central server is honest-but-curious, meaning it strictly follows the federated aggregation protocol but may attempt to infer sensitive local data from the uploaded model updates. Therefore, raw data is strictly retained locally to prevent direct privacy leakage. Regarding the clients, we assume a fraction of the participating nodes may act as malicious participants or become compromised clients. These adversaries aim to degrade the global model performance or inject targeted backdoors by submitting manipulated local gradients or poisoned model updates during the federated training process. Furthermore, we consider external adversaries who attempt to evade detection at the inference stage. These attackers possess the ability to inject constrained adversarial perturbations into the input sensing signals, such as slightly modifying access frequencies or disguising abnormal transaction requests, thereby creating anomaly camouflage to bypass the local detection boundaries.

Beyond merely resisting these malicious updates and input perturbations, modern cyberattack detection in distributed environments necessitates a governance-aware perspective. As emphasized in recent studies on explainable cyberattack detection and regulatory compliance [72], robust anomaly detection should not only optimize classification accuracy but also provide transparent, auditable, and operationally meaningful outputs. When compromised clients or external adversaries attempt to manipulate the system, the integration of temporal and feature attention mechanisms in our local representation module ensures that the decision-making process remains interpretable. This explainability is crucial for security analysts to trace the root cause of anomalous decisions, verify whether an alert stems from legitimate system faults or adversarial evasion, and ensure that the collaborative security monitoring process complies with strict data governance and regulatory auditing standards.

### 3.4. Proposed Method

#### 3.4.1. Overall

A privacy-preserving federated deep anomaly detection framework is proposed for distributed security sensing systems. The overall workflow starts from processed multidimensional security sensing sequences, for which temporal alignment, normalization, window segmentation, and label construction have already been completed. For an arbitrary client, the local input is represented as a security-state sequence composed of consecutive time windows, where each time step contains multiple categories of features, including system load, network traffic, access behavior, service requests, terminal status, and anomaly alarms. The input sequence first enters the local deep security sensing representation module on the client side, where the original multidimensional features are mapped into a unified latent space to mitigate the effects caused by differences in feature scales and data sources. Subsequently, a temporal encoder is employed to model state variations across consecutive windows, thereby capturing short-term anomalous fluctuations, long-term trend shifts, and coupling relationships among multiple variables. To enable the model to focus more strongly on key factors highly associated with anomaly risks, the encoded hidden features are further processed by temporal attention and feature attention mechanisms. As a result, high-risk signals such as sudden response-latency increases, elevated failure-request ratios, abnormal network connections, concentrated login failures, and abnormal device loads are assigned higher weights in the final representation. After this module, each client obtains a local security sensing representation, which is then fed into the anomaly detection head to output normal or abnormal probabilities, risk scores, and specific attack-category predictions. To adapt to differences in business patterns, anomaly proportions, and device states among different clients, the model is divided into a shared security sensing encoder and a local personalized detection head. The shared encoder is used to learn general anomaly patterns across nodes, whereas the local detection head preserves client-specific distribution characteristics and decision boundaries. During local training, the detection loss is optimized based on local samples, while personalized constraints and representation-consistency constraints are introduced so that the local model can adapt to its own data without excessively deviating from the global security knowledge space. To enhance input-level robustness, lightweight adversarial perturbation samples are further constructed during local training. The original samples and perturbed samples are jointly fed into the same encoder and detection head, and the normal detection loss and robust detection loss are jointly optimized. Consequently, stable decisions can still be maintained when noise injection, feature masking, and anomaly camouflage occur. After local updates are completed, only the parameter changes of the shared encoder are uploaded by each client, whereas the original local data and personalized detection head are not uploaded. After receiving model updates from different clients, the server does not perform simple averaging. Instead, client credibility is estimated according to update-direction consistency, parameter variation magnitude, historical contribution stability, and validation-loss changes. Stable and beneficial updates are assigned higher weights, whereas updates with excessively large magnitudes or abnormal directions are down-weighted or filtered. Subsequently, a new global shared encoder is generated through trust-aware weighted robust aggregation and is distributed back to the clients for the next training round. Through the iterative process of “local representation learning–personalized detection–adversarially robust training–trust-aware federated aggregation–global model redistribution”, collaborative modeling of multi-node security knowledge is achieved without sharing raw data, while both distribution-heterogeneity adaptation and malicious-perturbation defense are considered.

#### 3.4.2. Local Deep Security Sensing Representation Module

The local deep security sensing representation module is designed to extract anomaly-related representations from processed multidimensional temporal security sensing sequences within each client. For client *k*, the input sample is denoted as $X_{k}\in R^{T\times d}$, where *T* denotes the length of the time window and *d* denotes the dimension of security sensing features at each time step. These features may include system load, response latency, network traffic, login behavior, terminal status, and service-request statistics. To map features from different sources, scales, and statistical meanings into a unified representation space, a linear embedding layer is first adopted to perform feature projection, yielding $E_{k}=X_{k}W_{e}+b_{e}$, where $W_{e}\in R^{d\times h}$, $b_{e}$ denotes the bias term, and *h* denotes the latent-space dimension. This step converts the original security variables into unified high-dimensional semantic representations, making it easier for subsequent networks to learn correlations among different sensing signals. Subsequently, to capture both local anomalous fluctuations and long-term state dependencies, a hybrid temporal structure composed of one-dimensional convolution, a gated temporal encoder, and a self-attention layer is adopted. Specifically, the one-dimensional convolution layer extracts local patterns along the temporal dimension from the embedded sequence, which can be expressed as $C_{k}=\sigma \left(\mathrm{Conv}1D\left(E_{k};W_{c}\right)+b_{c}\right)$, where the convolution kernels are used to perceive short-term changes such as request-frequency bursts, response-latency jumps, increased failure rates, and abnormal network connections within adjacent time windows. Compared with directly using recurrent networks, the convolutional structure can capture local abrupt changes more efficiently and reduce the interference of short-term noise in overall sequence modeling.

**Figure 1:**
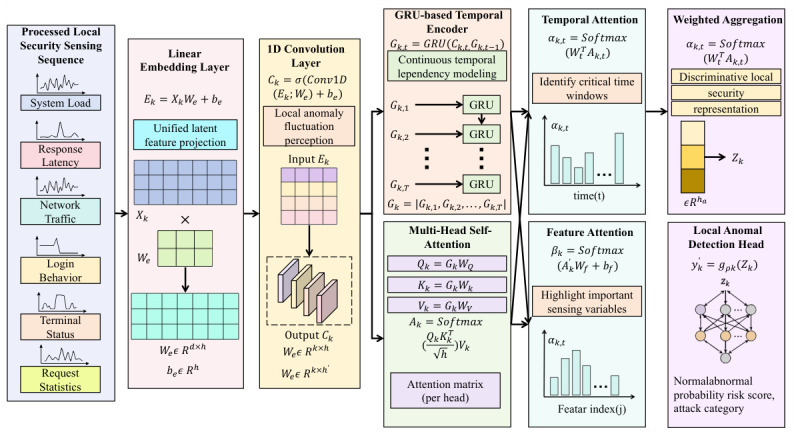
The figure illustrates the overall workflow of the local deep security sensing representation module, where multisource security sensing sequences are processed through linear embedding, 1D convolution, GRU-based temporal encoding, multi-head self-attention, and temporal–feature attention-based weighted aggregation to generate discriminative local security representations for anomaly detection.

As shown in Figure 1, after local temporal features are obtained, $C_{k}$ is further fed into a gated recurrent encoder to model the continuous dependencies of security states evolving over time. Its hidden state can be represented as $G_{k,t}=\mathrm{GRU}\left(C_{k,t},G_{k,t-1}\right)$, where the gating mechanism controls the retention and forgetting of historical information through update gates and reset gates. This enables the model to distinguish normal periodic business fluctuations from the continuous accumulation of abnormal states. For example, a short-term increase in transaction volume may correspond to a normal business peak, whereas a continuous increase accompanied by failure rates, latency, and abnormal connections is more likely to indicate a potential attack. To further capture long-range dependencies and dynamic cross-feature associations, a multi-head self-attention mechanism is introduced on top of the recurrent encoding results. Specifically, $Q_{k}=G_{k}W_{Q}$, $K_{k}=G_{k}W_{K}$, and $V_{k}=G_{k}W_{V}$ are computed, and $A_{k}=\mathrm{Softmax}\left(Q_{k}K_{k}^{⊤}/\sqrt{h}\right)V_{k}$ is then obtained. This structure can adaptively allocate attention weights according to the correlations among different time steps, enabling the model to discover abnormal propagation relationships across distant windows, such as the potential association between early increases in login failures and subsequent abnormal transaction requests.

To enhance the sensitivity of the model to key anomalous signals, a joint temporal-attention and feature-attention mechanism is designed after the self-attention output. Temporal attention is used to measure the importance of different time steps for anomaly judgment, which can be expressed as $\alpha_{k,t}=\mathrm{Softmax}\left(w_{t}^{⊤}A_{k,t}\right)$. Feature attention is used to characterize the contribution of different security sensing variables to risk identification, which can be expressed as $\beta_{k}=\mathrm{Softmax}\left(W_{f}{\bar{A}}_{k}+b_{f}\right)$, where ${\bar{A}}_{k}$ denotes the aggregated representation along the temporal dimension. Finally, the local security sensing representation is obtained through weighted aggregation and is denoted as $z_{k}=\sum_{t=1}^{T}\alpha_{k,t}\left(A_{k,t}⊙\beta_{k}\right)$, where ⊙ denotes element-wise weighting. This design enables the model to answer two key questions simultaneously: in which time windows the anomaly mainly occurs and which security variables are most critical to the anomaly. Therefore, the discriminability and interpretability of the representation can be improved. Finally, $z_{k}$ is fed into the local anomaly detection head to obtain the prediction result ${\hat{y}}_{k}=g_{\phi_{k}}\left(z_{k}\right)$, and optimization is performed through the detection loss $L_{det}^{k}$. Since this module is deployed locally on the client side, raw data do not need to be uploaded to the server. Meanwhile, its shared encoding part can participate in federated aggregation, allowing common security patterns learned from different nodes to be transferred to the global model. Overall, through the sequential process of “feature embedding–local convolutional perception–gated temporal modeling–self-attention association learning–joint temporal and feature weighting”, this structure can effectively adapt to the high-dimensional, heterogeneous, time-varying, and anomaly-scarce characteristics of distributed security sensing data, thereby providing stable and discriminative local representations for subsequent personalized federated optimization and robust anomaly detection.

#### 3.4.3. Privacy-Preserving Federated Optimization and Personalized Aggregation Module

The privacy-preserving federated optimization and personalized aggregation module is located after the local security sensing representation module. It is designed to enable multi-node collaborative learning without exposing raw security sensing data and to mitigate the non-independent and identically distributed problem caused by differences in business load, anomaly ratio, device status, and attack type among clients. The complete model is decomposed into three parts: a security sensing encoder involved in federated sharing, a lightweight adaptation layer, and a local personalized detection head. The shared encoder is formed by the aforementioned temporal representation network, and its output feature dimension is denoted as *h*. A group of client adaptation layers is arranged after the shared encoder. This structure consists of $L_{a}$ fully connected transformations, where the input width of the *l*-th layer is $p_{l-1}$, the output width is $p_{l}$, and the parameters are $A_{k}^{l}\in R^{p_{l-1}\times p_{l}}$ and $a_{k}^{l}\in R^{p_{l}}$, with $p_{0}=h$. If the security sensing representation is regarded as a one-dimensional feature map, this adaptation layer can also be equivalently interpreted as a channel-mapping module. Its input height is 1, its width is *h*, and its number of channels is $c_{0}$. After the *l*-th layer, a node-specific representation with height 1, width $p_{l}$, and channel number $c_{l}$ is obtained. The personalized detection head consists of an $L_{p}$-layer local classification network, where the width of the *m*-th layer is $q_{m}$, and the output dimension of the final layer is the number of anomaly categories *C* or the binary risk-score dimension. The parameters of the shared encoder are denoted as Θ, while the parameters of the client adaptation layer and detection head are denoted as $\Psi_{k}$ and $\Phi_{k}$, respectively. During federated training, only Θ participates in server-side aggregation, whereas $\Psi_{k}$ and $\Phi_{k}$ are always retained locally on the client side. This prevents node-specific business patterns and label distributions from being leaked through the parameters of the model head.

**Figure 2:**
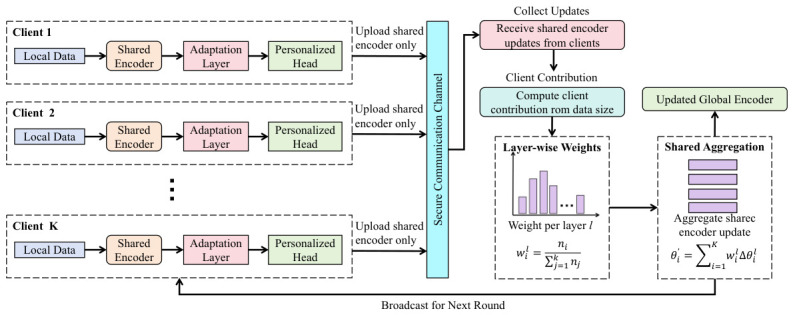
The figure illustrates the overall workflow of the privacy-preserving federated optimization and personalized aggregation module, where clients upload only shared encoder updates while retaining local adaptation layers and personalized heads, and the server generates the global encoder through client contribution modeling, layer-wise weight computation, and shared aggregation for the next training round.

As shown in Figure 2, for client *k*, the representation output by the local security sensing encoder is denoted as $r_{k}$, and the recursive computation of the adaptation layer can be written as:

(5) $$u_{k}^{l}=\rho \left(u_{k}^{l-1}A_{k}^{l}+a_{k}^{l}\right),\;l=1,\ldots ,L_{a},\;u_{k}^{0}=r_{k},$$

where ρ(·) denotes a nonlinear activation function. The personalized detection head further outputs the prediction distribution:

(6) $${\hat{y}}_{k}=\varpi \left(u_{k}^{L_{a}}B_{k}^{1}+b_{k}^{1},\ldots ,B_{k}^{L_{p}},b_{k}^{L_{p}}\right),$$

where ϖ(·) denotes the personalized discrimination process composed of multilayer local mappings and an output function, and $B_{k}^{m}$ and $b_{k}^{m}$ denote the parameters of the *m*-th detection-head layer. To enable the shared encoder to learn common cross-node security patterns while preventing excessive drift of local models under non-IID data, a local optimization objective containing detection risk, a personalization stability term, and a shared-space constraint is constructed:

(7) $$J_{k}\left(\Theta_{k},\Psi_{k},\Phi_{k}\right)=E_{\left(x,y\right)∼D_{k}}\ell \left(f_{k}\left(x;\Theta_{k},\Psi_{k},\Phi_{k}\right),y\right)+\mu \Omega_{k}\left(\Theta_{k},\bar{\Theta }\right)+\nu \Gamma_{k}\left(\Psi_{k},\Phi_{k}\right),$$

where ℓ(·) denotes the anomaly detection loss, $\bar{\Theta }$ denotes the global shared parameters distributed by the server, $\Omega_{k}\left(\cdot \right)$ is used to restrict local drift of the shared encoder, $\Gamma_{k}\left(\cdot \right)$ is used to constrain the complexity of the personalized layers, and μ and ν are balancing coefficients. Different from simple parameter averaging, a hierarchical aggregation strategy is further used to assign different weights to updates from different layers of the shared encoder. Let the parameters of the *s*-th shared layer be $\Theta^{s}$, and let the update uploaded by a client for this layer be $\Delta_{k}^{s}$. The server-side aggregation can then be expressed as:

(8) $${\bar{\Theta }}_{r+1}^{s}={\bar{\Theta }}_{r}^{s}+\sum_{k\in S_{r}}\omega_{k}^{s}\Delta_{k}^{s},\;s=1,\ldots ,L_{s},$$

where $L_{s}$ denotes the number of shared-encoder layers, and $\omega_{k}^{s}$ denotes the aggregation weight of client *k* at the *s*-th layer. To reflect the contribution of different client updates to the global model, $\omega_{k}^{s}$ is jointly determined by sample scale, update stability, and layer-wise similarity:

(9) $$\omega_{k}^{s}=\frac{\exp\left(\tau_{1}\chi_{k}+\tau_{2}\xi_{k}^{s}-\tau_{3}\zeta_{k}^{s}\right)}{\sum_{j\in S_{r}}\exp\left(\tau_{1}\chi_{j}+\tau_{2}\xi_{j}^{s}-\tau_{3}\zeta_{j}^{s}\right)},$$

where $\chi_{k}$ denotes the effective sample contribution of the client, $\xi_{k}^{s}$ denotes the consistency between the update of the *s*-th layer and the global descent direction, $\zeta_{k}^{s}$ denotes the abnormal magnitude of the layer-wise update, and $\tau_{1},\tau_{2},\tau_{3}$ are adjustment coefficients. Since $\omega_{k}^{s}$ is computed through a normalized exponential function, the following relationship holds:

(10) $$\omega_{k}^{s}>0,\;\sum_{k\in S_{r}}\omega_{k}^{s}=1.$$

Therefore, the new server-side model ${\bar{\Theta }}_{r+1}^{s}$ is a convex combination of shared-layer updates from different clients. If the client updates satisfy the bounded condition $∥\Delta_{k}^{s}∥\leq M_{s}$, the aggregated update satisfies:

(11) $$∥\sum_{k\in S_{r}}\omega_{k}^{s}\Delta_{k}^{s}∥\leq \sum_{k\in S_{r}}\omega_{k}^{s}∥\Delta_{k}^{s}∥\leq M_{s}.$$

This relationship indicates that, under the constraint of normalized hierarchical weights, the global update will not exceed the bounded range of client shared-layer updates, thereby reducing the damage caused by abnormally large updates to the global model. Furthermore, if the local objective function is α-smooth with respect to the shared parameters, the following inequality holds:

(12) $$J\left({\bar{\Theta }}_{r+1}\right)\leq J\left({\bar{\Theta }}_{r}\right)+\langle \nabla J\left({\bar{\Theta }}_{r}\right),\sum_{k\in S_{r}}\omega_{k}\Delta_{k}\rangle +\frac{\alpha }{2}{∥\sum_{k\in S_{r}}\omega_{k}\Delta_{k}∥}^{2}.$$

When the hierarchical weights increase the proportion of client updates consistent with the global descent direction, the inner-product term is more likely to be negative, making the aggregation direction closer to the global risk-reduction direction. Meanwhile, the local adaptation layers and detection heads do not participate in aggregation, allowing different clients to retain their own business boundaries, anomaly proportions, and device-operation patterns. For the distributed security sensing task considered in this study, this module provides several advantages. The server can only observe limited parameter updates from the shared encoder and cannot directly obtain raw information such as transaction records, access behaviors, and device states. The shared hierarchical aggregation mechanism can learn common anomaly representations across nodes. The personalized adaptation layers can absorb local differences caused by non-IID distributions. The bounded convex-combination aggregation improves training stability, allowing the model to achieve better generalization and privacy protection in heterogeneous scenarios such as financial security, edge gateways, and multi-terminal state monitoring.

#### 3.4.4. Adversarially Robust Security Detection and Trust-Aware Aggregation Module

The adversarially robust security detection and trust-aware aggregation module is located after local security sensing representation learning and federated collaborative optimization. It is designed to improve the security stability of the model at both the input perturbation level and the federated update level. On the client side, this module consists of an adversarial perturbation generation unit, a robust feature-consistency constraint unit, and an anomaly detection output layer. On the server side, it consists of an update-statistic modeling unit, a client trustworthiness evaluation unit, and a trust-aware weighted aggregation unit.

**Figure 3:**
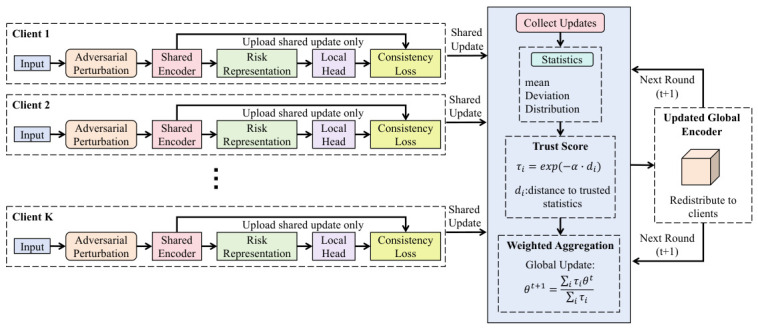
The figure illustrates the overall workflow of the adversarially robust security detection and trust-aware aggregation module, where each client enhances local robustness through adversarial perturbation, shared encoding, risk representation, local detection, and consistency constraints, while only shared updates are uploaded and aggregated by the server using statistical analysis, trust scoring, and weighted aggregation to generate the global encoder for the next training round.

As shown in Figure 3, for client *k*, the sequence representation obtained by the local security sensing encoder is denoted as $R_{k}\in R^{T\times h}$, where *T* denotes the time-window length and *h* denotes the latent-space width. The anomaly detection output layer adopts a local discriminative network composed of $L_{o}$ fully connected mappings. The input width of the *m*-th layer is denoted as $o_{m-1}$, the output width is denoted as $o_{m}$, and the corresponding parameters are $U_{k}^{m}\in R^{o_{m-1}\times o_{m}}$ and $c_{k}^{m}\in R^{o_{m}}$. The final layer outputs either the number of anomaly categories *C* or the risk-score dimension. If the input representation is regarded as a one-dimensional temporal feature map, the input height of this module is *T*, the width is *h*, and the number of channels is $c_{r}$. After attention pooling, it is compressed into a risk representation with height 1, width *h*, and channel number $c_{z}$, which is then fed into the local discriminative network to generate the anomaly probability distribution. To simulate evasive modifications of security sensing signals by attackers, constrained perturbations are constructed in both the local input space and the latent space. For an input sample $X_{k}$, the perturbation direction is determined by the gradient of the current detection loss with respect to the input, and the perturbation range is constrained by a magnitude projection function:

(13) $${\tilde{X}}_{k}=\Pi_{B(X_{k},\varepsilon )}\left(X_{k}+\varepsilon \cdot \frac{\nabla_{X_{k}}L_{cls}^{k}}{∥\nabla_{X_{k}}L_{cls}^{k}∥+\delta }\right),$$

where ${\tilde{X}}_{k}$ denotes the perturbed security sensing sequence, $\Pi_{B(X_{k},\varepsilon )}\left(\cdot \right)$ denotes the projection of the perturbed sample into a feasible domain centered at $X_{k}$ with radius controlled by ε, and δ is used to avoid numerical instability caused by an excessively small gradient norm. This design can simulate attack forms such as slight response-latency modification, local request-frequency disturbance, traffic-statistics noise injection, and access-behavior camouflage, while ensuring that the perturbed samples remain within a reasonable security sensing feature range. To avoid robustness being obtained only at the input level, a consistency constraint is further introduced at the latent representation level. Let the representations of the original sample and the perturbed sample after passing through the shared encoder be $z_{k}$ and ${\tilde{z}}_{k}$, respectively. The robust consistency loss is defined as:

(14) $$L_{con}^{k}=1-\frac{z_{k}^{⊤}{\tilde{z}}_{k}}{∥z_{k}∥∥{\tilde{z}}_{k}∥}.$$

This term requires the model to maintain similar risk-semantic representations when the input is slightly perturbed, thereby reducing the influence of anomaly camouflage on the decision boundary. The overall robust objective function on the client side can be expressed as:

(15) $$L_{adv}^{k}=L_{cls}^{k}\left(X_{k},y_{k}\right)+\lambda_{a}L_{cls}^{k}\left({\tilde{X}}_{k},y_{k}\right)+\lambda_{c}L_{con}^{k}+\lambda_{r}{∥\Theta_{k}∥}_{2}^{2},$$

where the first term ensures the detection capability of the model on original samples, the second term improves the recognition capability for perturbed samples, the third term constrains the consistency between the original and perturbed representations, and the fourth term limits model complexity. This objective shows that the model no longer merely learns a classification boundary under empirical risk minimization, but instead learns a stable risk boundary within a local perturbation neighborhood. Its robust optimization objective can be further understood as:

(16) $$\min_{\Theta_{k},\Phi_{k}}E_{\left(X_{k},y_{k}\right)∼D_{k}}\left[\max_{\Delta X_{k}\in B\left(0,\varepsilon \right)}\ell \left(f_{k}\left(X_{k}+\Delta X_{k}\right),y_{k}\right)\right].$$

This expression indicates that the local model is required to maintain a low detection loss even under the most unfavorable perturbation conditions, making it more suitable for financial security sensing scenarios in which anomalous signals may be deliberately weakened, camouflaged, or mixed with normal fluctuations. On the server side, the trust-aware aggregation mechanism is used to prevent malicious clients from affecting the global shared encoder by uploading abnormal updates. The shared-parameter update uploaded by client *k* in round *r* is denoted as $\Delta \Theta_{k}^{r}$. The server first computes direction consistency, magnitude stability, and historical reliability, and then forms a trustworthiness score. Direction consistency is used to measure whether the current update is close to the common descent direction of the majority of clients. Magnitude stability is used to penalize abnormally large parameter variations, and historical reliability is used to characterize the contribution stability of the client across previous training rounds. The trustworthiness score can be expressed as:

(17) $$s_{k}^{r}=\alpha_{1}\frac{\langle \Delta \Theta_{k}^{r},{\bar{\Delta \Theta }}^{r}\rangle }{∥\Delta \Theta_{k}^{r}∥∥{\bar{\Delta \Theta }}^{r}∥}-\alpha_{2}\left|∥\Delta \Theta_{k}^{r}∥-\mathrm{Med}\left(\{∥\Delta \Theta_{j}^{r}{∥\}}_{j\in S_{r}}\right)\right|+\alpha_{3}h_{k}^{r},$$

where ${\bar{\Delta \Theta }}^{r}$ denotes the reference update direction in the current round, Med(·) denotes the median statistic of client update norms, and $h_{k}^{r}$ denotes the historical stability record of the client. Subsequently, the server maps the trustworthiness score into a normalized aggregation weight:

(18) $$\pi_{k}^{r}=\frac{\exp(s_{k}^{r})}{\sum_{j\in S_{r}}\exp\left(s_{j}^{r}\right)}.$$

Finally, the global shared encoder is updated through trust-aware weighting:

(19) $$\Theta_{g}^{r+1}=\Theta_{g}^{r}+\sum_{k\in S_{r}}\pi_{k}^{r}\Delta \Theta_{k}^{r}.$$

Since $\pi_{k}^{r}>0$ and $\sum_{k\in S_{r}}\pi_{k}^{r}=1$, the global update is a convex combination of client updates. When malicious clients upload reversed gradients, random perturbations, or abnormally large updates, their direction consistency is lower and their magnitude deviation is larger, causing their corresponding trust-aware weights to be suppressed. If normal client updates satisfy $∥\Delta \Theta_{k}^{r}∥\leq M$, and abnormal clients are assigned smaller weights by the trustworthiness function, the global abnormal-offset term satisfies:

(20) $$∥\sum_{k\in A_{r}}\pi_{k}^{r}\Delta \Theta_{k}^{r}∥\leq \sum_{k\in A_{r}}\pi_{k}^{r}∥\Delta \Theta_{k}^{r}∥,$$

where $A_{r}$ denotes the set of potential abnormal clients. This inequality indicates that a lower trustworthiness weight leads to a more restricted influence of malicious updates on the global model. For the task considered in this study, this module provides several advantages. Local adversarial training can improve tolerance to input noise, anomaly camouflage, and feature perturbations. The representation-consistency constraint can maintain the stability of the risk-semantic space, and server-side trust-aware aggregation can suppress the damage caused by malicious clients or abnormal nodes to the global model. Through the joint design of input-level robust learning and federated-level trust-aware aggregation, the proposed method can better adapt to practical distributed security sensing systems characterized by complex attack sources, scarce anomaly patterns, uneven client quality, and heterogeneous data distributions.

## 4. Results and Discussion

### 4.1. Experimental Configuration

#### 4.1.1. Hardware and Software Platform

The experiments were conducted on a high-performance deep learning server. The hardware configuration was equipped with dual Intel Xeon Gold 6240R processors (24 cores and 48 threads per CPU), an NVIDIA RTX 4090 GPU with 24 GB of memory, 256 GB DDR4 memory, and a 4 TB NVMe SSD for data storage.

The software environment was based on the Ubuntu 22.04 LTS 64-bit operating system. Model training and optimization were implemented using Python 3.10 and PyTorch 2.1.0, supported by CUDA 12.1 and cuDNN 8.9 for GPU acceleration. The federated learning components were built using Flower 1.5.0. Data preprocessing and metric calculations were performed with NumPy 1.26.0, Pandas 2.1.1, and Scikit-learn 1.3.1. Adversarial sample generation and robust training modules were implemented using TorchAttack 3.3.0 and Foolbox 3.3.3, while TensorBoard 2.14.0, Matplotlib 3.8.0, and Seaborn 0.13.0 were utilized for logging and visualization.

In the experimental setting, the multi-node security sensing dataset was first partitioned. To ensure that the training process could sufficiently learn anomaly patterns while avoiding data leakage during testing, the dataset was split according to temporal order and client independence. Specifically, the data partitioning strictly followed a device-level separation strategy rather than random client separation. Each client naturally corresponds to a specific physical node or institutional boundary, thereby inherently constructing a non-IID data distribution where each client contains different anomaly categories, feature characteristics, and data volumes based on its operational role. Within each client, 70% of the data were used for model training, 15% were used for validation-based hyperparameter tuning and model selection, and the remaining 15% were used for final testing. Since the target scenario of this study was federated learning, each client independently performed training, validation, and test partitioning to preserve the data isolation characteristics of a real distributed environment. To further improve the stability and credibility of the experimental results, a 5-fold cross-validation strategy was adopted. The dataset was divided into 5 non-overlapping subsets, one subset was selected as the test set each time, and the remaining 4 subsets were used as training and validation data. The final experimental results were reported as the average values over the 5 runs, thereby reducing result fluctuations caused by random data partitioning.

During model training, the AdamW optimizer was adopted to optimize the deep security sensing model. The initial learning rate was set to $\eta =1\times 10^{-4}$, and the weight decay coefficient was set to $\lambda =1\times 10^{-5}$ to reduce the risk of overfitting. The batch size was set to batch_size=64; the number of local client training epochs was set to E=5, and the maximum number of global federated communication rounds was set to R=100. For the federated learning protocol, the global aggregation frequency was set to once every 5 local epochs. The client participation ratio was set to 1.0, meaning all ten clients participated in each communication round to ensure comprehensive global knowledge aggregation across the highly skewed non-IID nodes. Furthermore, an early stopping criterion was implemented, which halted the federated training process if the global validation loss did not improve for 15 consecutive communication rounds, thereby preventing overfitting and reducing unnecessary communication overhead. For temporal input data, the sliding window length was set to T=32, a parameter choice formulated to strike an optimal balance between capturing sufficient long-range temporal dynamic patterns and controlling local computational complexity. In the Transformer encoder, the hidden dimension was set to d=128, the number of multi-head attention heads was set to h=8, the feed-forward network dimension was set to 512, and the Dropout ratio was set to p=0.2 to improve generalization capability under complex temporal fluctuations.

During federated learning optimization, the personalized aggregation coefficient was set to α=0.5 to alleviate client drift caused by non-IID data and to balance the contributions of global shared parameters and local personalized parameters. In the robust aggregation stage, the client credibility weight update parameter was set to β=0.3, and the abnormal update filtering threshold was set to τ=0.15 to reduce the influence of abnormal gradients uploaded by malicious clients on the global model. For the adversarial training module, the input perturbation intensity was set to ϵ=0.01, and the adversarial loss weight was set to γ=0.2, thereby enhancing robustness against input noise, anomaly camouflage, and evasion attacks. During data augmentation, the standard deviation of Gaussian noise was set to σ=0.05, the random masking ratio was set to 0.1, and the random temporal offset range was set to [−3,3] to simulate sensor errors, log missingness, and temporal fluctuations in real distributed security sensing environments.

#### 4.1.2. Baseline Models and Evaluation Metrics

Three categories of representative methods were selected as baselines for comparative analysis, including Centralized Training [73], Local Training [74], LSTM [75], GRU [76], Transformer [77], Autoencoder [78], Isolation Forest [79], FedAvg [74], FedProx [80], SCAFFOLD [81], FedNova [82], MOON [83], Median Aggregation [84], Trimmed Mean [85], Krum [84], and Robust FedAvg [86]. To ensure a fair and rigorous comparative analysis, all baseline models were evaluated under strictly comparable settings. Specifically, every method utilized the exact same preprocessed input features, data partitioning splits, and global random seeds. Hyperparameters for each baseline were optimally tuned using the identical validation set strategy to prevent evaluation bias. Furthermore, to account for experimental variance and demonstrate statistical stability, all performance metrics are reported as the average values accompanied by their standard deviations across multiple independent runs. In the context of our baseline selection and experimental design, recent advancements in federated intrusion detection were also thoroughly considered. For instance, the integration of federated learning with local data preservation and temporal feature extraction has shown significant promise for zero-trust intrusion detection in distributed networks [87]. Inspired by such frameworks, our baseline selection covers a wide spectrum of modeling strategies. Among centralized and local training methods, Centralized Training performs unified training by aggregating all client data, thereby achieving strong global feature learning capability and typically stable model performance. Local Training independently trains a model using only local node data, which avoids privacy leakage caused by data sharing. LSTM learns long-term sequential dependencies through gated memory mechanisms and is suitable for modeling complex security behaviors. GRU reduces network complexity while retaining temporal modeling capability, resulting in relatively high training efficiency. Transformer captures global temporal correlations through self-attention mechanisms and exhibits strong modeling capability for long-range anomaly dependencies. Autoencoder performs anomaly detection based on reconstruction errors and can effectively learn normal behavioral patterns. Isolation Forest detects outlier samples based on a random partitioning strategy and has relatively low computational complexity. Among classical federated learning methods, FedAvg performs global model aggregation through weighted parameter averaging and has the advantages of structural simplicity and high communication efficiency. FedProx alleviates client drift under non-IID scenarios by introducing a proximal constraint. SCAFFOLD improves federated training stability by using control variates to correct local update bias. FedNova reduces the impact of different local training steps among clients through normalized local updates. MOON introduces a model contrastive learning mechanism to enhance global model consistency. Among robust federated learning methods, Median Aggregation reduces the influence of abnormal gradients through parameter median aggregation and provides strong resistance to abnormal updates. Trimmed Mean suppresses malicious client interference by truncating extreme parameters. Krum selects the most reliable client update based on parameter distances, thereby improving aggregation robustness. Robust FedAvg enhances model stability under poisoning attacks by incorporating robust statistical strategies into traditional FedAvg.

For the distributed security anomaly detection task, Accuracy, Precision, Recall, F1-score, AUC, and PR-AUC were used as the main evaluation metrics. Accuracy was used to measure the overall classification correctness of the model. Precision was used to evaluate the proportion of true anomalies among the samples predicted as anomalous. Recall was used to measure the detection capability of the model for anomalous samples. F1-score comprehensively reflected the balance between Precision and Recall. AUC was used to describe the ability of the model to distinguish normal and anomalous samples under different thresholds. PR-AUC focused more on detection performance under the condition of scarce anomalous samples. The calculation formulas for each evaluation metric are as follows:

(21) $$Accuracy=\frac{TP+TN}{TP+TN+FP+FN},$$

(22) $$Precision=\frac{TP}{TP+FP},$$

(23) $$Recall=\frac{TP}{TP+FN},$$

(24) $$F1=2\times \frac{Precision\times Recall}{Precision+Recall},$$

(25) $$AUC=\int_{0}^{1}TPR\left(FPR\right)d\left(FPR\right),$$

(26) $$PR-AUC=\int_{0}^{1}Precision\left(Recall\right)d\left(Recall\right),$$

where TP denotes the number of true anomalous samples correctly detected, TN denotes the number of true normal samples correctly identified, FP denotes the number of normal samples misclassified as anomalous, and FN denotes the number of anomalous samples misclassified as normal. TPR denotes the true positive rate, namely the anomaly detection rate, and FPR denotes the false positive rate, namely the false alarm rate for normal samples. Precision and Recall correspond to the coordinate values in the precision–recall curve.

### 4.2. Comparison of Anomaly Detection Performance

This experiment was designed to evaluate the effectiveness of the proposed method from the perspective of overall anomaly detection performance in distributed security sensing scenarios. The detection capability of different models was examined under high-dimensional temporal security signals, scarce anomaly samples, heterogeneous client distributions, and coupled multisource features.

**Figure 4:**
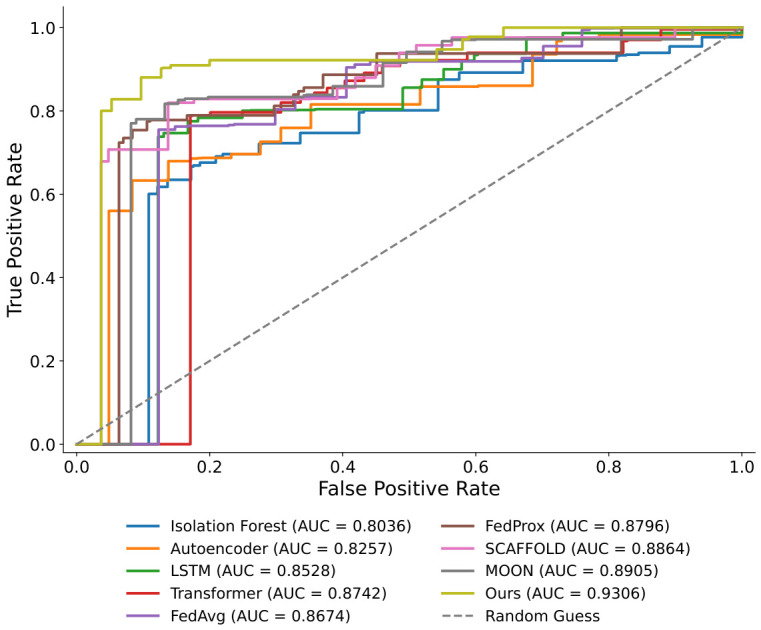
The ROC curves illustrate the classification discrimination capability of different anomaly detection methods in the distributed security sensing task.

As shown in Table 3 and Figure 4, Isolation Forest, as a conventional unsupervised anomaly detection method, achieved only 82.41%, 73.87%, and 72.15% in Accuracy, F1-score, and PR-AUC, respectively, yielding the lowest overall performance. This indicates that shallow anomaly discrimination based on isolation-based partitioning is insufficient for capturing temporal dependencies and nonlinear risk patterns in complex security sensing sequences. Autoencoder achieved a moderate improvement over Isolation Forest, with an F1-score of 76.49%, suggesting that reconstruction errors can reflect certain anomalous deviations. However, its optimization objective mainly focuses on input reconstruction rather than anomaly discrimination, which limits its ability to identify stealthy attacks and minority anomaly classes. The performance of LSTM and Transformer was further improved. In particular, Transformer achieved an F1-score and AUC of 82.17% and 87.42%, respectively, outperforming LSTM, whose corresponding values were 79.77% and 85.28%. As visually corroborated by the ROC curves in Figure 4, the curve corresponding to Transformer exhibits a noticeably sharper ascent toward the upper-left quadrant compared to recurrent architectures. This visual evidence further substantiates that the self-attention mechanism is more effective than recurrent structures in modeling long-range temporal dependencies and dynamic associations among multiple variables, providing distinct advantages in identifying cross-time security signals such as response latency variations, traffic bursts, and abnormal access behaviors. By constructing tighter decision boundaries, the proposed attention-based architecture successfully isolates sparse anomaly signals from benign background traffic, which directly translates to the superior area under the curve observed in the figure.

From the perspective of federated learning methods, FedAvg obtained an F1-score of 80.99%, which was lower than that of Transformer. Although FedAvg can exploit multi-client data for collaborative training, simple parameter averaging is prone to client drift under non-IID distributions, making it difficult for the global model to accommodate local anomaly patterns across different nodes. FedProx alleviated local model deviation through a proximal constraint and improved all metrics compared with FedAvg, achieving an F1-score of 82.27%. SCAFFOLD further corrected client gradient bias using control variates, increasing Recall and F1-score to 81.57% and 82.95%, respectively. MOON enhanced the consistency between global and local representations through a model-contrastive constraint and achieved the best performance among the federated baselines, with an F1-score and PR-AUC of 83.72% and 82.97%, respectively. In contrast, the proposed method achieved 92.37%, 88.26%, 88.83%, 93.06%, and 89.15% in Accuracy, Recall, F1-score, AUC, and PR-AUC, respectively, clearly outperforming all baselines. This improvement can be attributed to the fact that the proposed method does not merely perform global parameter averaging. Instead, anomaly-related temporal representations are extracted through a local deep security sensing encoder, client-specific distribution boundaries are preserved through personalized detection heads, and the influence of abnormal updates is reduced through trust-aware aggregation. From the perspective of mathematical optimization, this design simultaneously reduces local empirical risk, client-drift errors, and aggregation bias caused by abnormal updates, thereby achieving more stable overall performance in distributed, security-sensitive, and non-IID anomaly detection tasks.

### 4.3. Robustness Comparison

This experiment was designed to evaluate the robust anomaly detection capability of different federated learning methods under input perturbations and malicious client attacks. All results in the table are reported using the F1-score, which more directly reflects the comprehensive recognition performance of models under scarce anomaly samples and class-imbalanced conditions.

**Figure 5:**
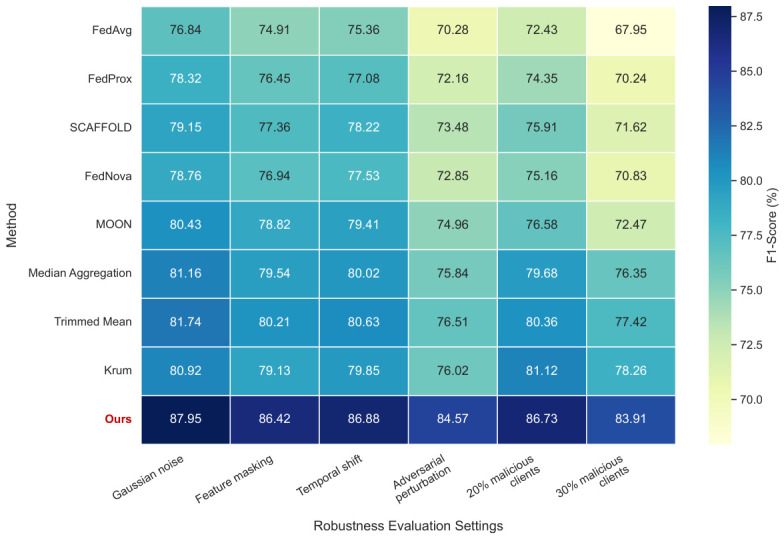
The heatmap compares the F1-scores of different federated learning and robust aggregation methods under input perturbations and malicious client attacks.

As shown in Table 4 and Figure 5, FedAvg showed the weakest performance across all perturbation scenarios, achieving only 76.84%, 74.91%, 75.36%, and 70.28% under Gaussian noise, feature masking, temporal shift, and adversarial perturbation, respectively. Its performance further decreased to 72.43% and 67.95% under 20% and 30% malicious clients. This extreme vulnerability is intuitively reflected in the performance heatmap in Figure 5, where the color gradients for FedAvg shift dramatically toward cooler and darker tones as the attack intensity increases, visually highlighting its extreme sensitivity to input noise, feature missingness, and malicious model updates. FedProx achieved a slight improvement over FedAvg, reaching 70.24% under 30% malicious clients, mainly because the proximal constraint can restrict excessive deviation of local models from the global model. However, malicious updates are not explicitly identified, and thus its defensive capability remains limited. SCAFFOLD and FedNova mitigated client drift through control-variate correction and normalized local updates, respectively, and their overall performance was slightly better than that of FedProx. Nevertheless, clear performance degradation was still observed under strong adversarial perturbations and high proportions of malicious clients. MOON achieved higher results under different perturbations, such as 80.43% under Gaussian noise and 74.96% under adversarial perturbation, indicating that representation contrastive constraints help improve feature-space stability. However, as the heatmap visually confirms, MOON mainly addresses representation deviation between global and local models and provides insufficient direct suppression against malicious client attacks, whereas the proposed method maintains stable and warmer color bands across all extreme adversarial scenarios.

Compared with standard federated learning methods, robust aggregation methods such as Median Aggregation, Trimmed Mean, and Krum showed more stable performance under malicious-client settings. In particular, Krum achieved 81.12% and 78.26% under 20% and 30% malicious clients, respectively, outperforming Median Aggregation and Trimmed Mean, which suggests that distance-based update selection can filter abnormal updates to some extent. Trimmed Mean performed slightly better under input perturbation scenarios, reaching 80.21% and 80.63% under feature masking and temporal shift, respectively. This is because trimmed averaging can weaken the influence of extreme parameter updates on the global model, although it does not directly enhance the robust representation capability of local input features. The proposed method achieved the best results in all scenarios, reaching 87.95%, 86.42%, 86.88%, 84.57%, 86.73%, and 83.91% under Gaussian noise, feature masking, temporal shift, adversarial perturbation, 20% malicious clients, and 30% malicious clients, respectively. From the perspective of mathematical optimization, conventional federated methods mainly minimize empirical risk on clean samples, whereas the proposed method extends the optimization objective to the input perturbation neighborhood through adversarial training, enabling the model to learn smoother and more stable anomaly decision boundaries. Meanwhile, trust-aware weighted aggregation dynamically adjusts client weights according to update-direction consistency, magnitude stability, and historical contribution, thereby reducing the interference of abnormal gradients with the global descent direction. Therefore, this module can simultaneously resist input-side perturbations and training-side poisoning, making it more suitable for distributed security sensing systems with complex attack sources, uneven client quality, and dynamically evolving anomaly patterns.

### 4.4. Ablation Study

This ablation experiment was designed to verify the individual contributions of the key components in the proposed privacy-preserving federated deep anomaly detection framework and to analyze whether the performance improvement of the full model results from the collaborative effect of multiple mechanisms.

**Figure 6:**
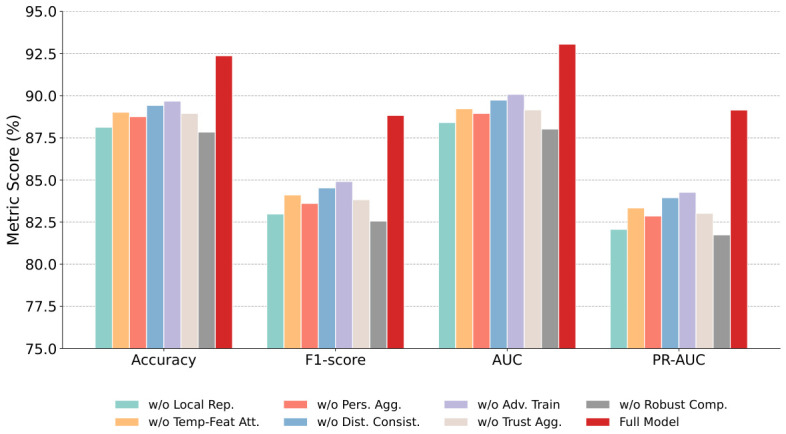
The bar chart compares different ablation variants and the full model in terms of Accuracy, F1-score, AUC, and PR-AUC, validating the contributions of local representation learning, temporal–feature attention, personalized aggregation, adversarial training, and trust-aware aggregation to the overall performance.

As shown in Table 5 and Figure 6, the ablation study provides a comprehensive quantitative and visual demonstration of the contribution of each proposed module. After removing the local deep security sensing representation module, Accuracy, Recall, F1-score, and PR-AUC decreased to 88.13%, 81.74%, 82.98%, and 82.07%, respectively. As illustrated in the bar chart in Figure 6, this variant exhibits one of the most substantial drops in bar height, visually emphasizing that temporal dependencies, multivariate coupling, and local anomalous fluctuations in high-dimensional security sensing signals cannot be effectively modeled by shallow feature mapping alone. After removing the Temporal–Feature Attention Mechanism, the F1-score was reduced to 84.11%, showing a clear downward trend in the visual representation compared with the full model. This indicates that, although the basic temporal encoder can still extract certain anomaly patterns, the absence of temporal and feature-wise weighted selection makes it difficult for the model to highlight key risk signals such as response latency spikes, concentrated login failures, and abnormal traffic fluctuations. After removing the Personalized Aggregation Module, the F1-score decreased to 83.61%, generating a noticeably lower performance bar. This suggests that substantial differences exist among clients in terms of business patterns and anomaly proportions in distributed security sensing scenarios. If all clients are forced to share the same decision boundary, the global model may become insufficiently adapted to local nodes. Ultimately, the bar chart effectively illustrates that the full model achieves compounded synergistic gains only when all architectural components are seamlessly integrated.

Furthermore, after removing the Distribution Consistency Constraint, the F1-score and PR-AUC decreased to 84.53% and 83.95%, respectively, indicating that this constraint can stabilize the shared representation space under non-IID conditions and prevent excessive deviation among security sensing features learned by different clients. After removing Adversarial Training, the F1-score was 84.91%, suggesting that the model still maintained certain detection capability on clean samples, but its adaptability to input perturbations, anomaly camouflage, and feature noise was weakened. After removing the Trust-aware Aggregation Module, the F1-score decreased to 83.83%, demonstrating that standard aggregation is vulnerable to abnormal clients or highly biased updates, which can destabilize the global descent direction. After removing all robust components, the model performance further decreased to an F1-score of 82.56% and a PR-AUC of 81.74%, confirming the complementary effects of adversarial training and trust-aware aggregation at the input layer and federated update layer. The full model achieved the best results across all metrics, with Accuracy, Recall, F1-score, AUC, and PR-AUC reaching 92.37%, 88.26%, 88.83%, 93.06%, and 89.15%, respectively. From the perspective of mathematical properties, the local representation module reduces nonlinear fitting errors in the feature space, the attention mechanism increases the discriminative weights of anomaly-related variables, personalized aggregation reduces optimization bias caused by client distribution shifts, the distribution consistency constraint compresses cross-node representation discrepancies, adversarial training smooths the decision boundary within local perturbation neighborhoods, and trust-aware aggregation weakens the interference of abnormal updates with the global optimization direction. Therefore, the performance improvement of the proposed method does not originate from a single component, but from the systematic gains produced by representation learning, personalized federated optimization, and robust security aggregation.

### 4.5. Sensitivity Analysis of Trust-Score Components

This experiment aims to systematically evaluate the sensitivity of the trust-aware aggregation mechanism to its core hyperparameters, specifically the regulatory coefficients $\alpha_{1}$, $\alpha_{2}$, and $\alpha_{3}$. These coefficients control the relative influence of update-direction consistency, magnitude deviation, and historical reliability within the trust-score calculation. The evaluation was conducted under a simulated attack scenario containing 20% malicious clients. To isolate the effect of each component, we systematically varied one coefficient across a predefined range while fixing the remaining two at their default optimal values ($\alpha_{1}=1.0$, $\alpha_{2}=1.0$, $\alpha_{3}=0.5$). The overall anomaly detection performance was recorded using the F1-score, complete with standard deviations from multiple independent runs, to observe the impact of hyperparameter fluctuations on global model robustness, as shown in Table 6.

The experimental results indicate that the proposed framework maintains high performance across a reasonable range of parameter values, demonstrating the inherent stability of the trust-aware aggregation design. Specifically, assigning an excessively low value to $\alpha_{1}$ significantly degrades robustness, as the server fails to adequately penalize malicious gradients that deviate from the global optimization direction. Conversely, setting $\alpha_{2}$ too high overly restricts normal magnitude fluctuations caused by the non-IID nature of the local client data, which inadvertently hinders the convergence of benign nodes. The historical reliability coefficient $\alpha_{3}$ serves as a critical stabilizing factor during the aggregation process. These empirical observations align closely with recent findings in reputation-based client selection studies [88], which highlight the absolute necessity of incorporating continuous historical contribution weighting to mitigate advanced federated attacks under distributed non-IID conditions.

### 4.6. Extended Adversarial Robustness Testing

To further validate the comprehensive defensive capabilities of the proposed framework against targeted manipulation and advanced model poisoning, we extended the robustness evaluation to explicitly include label-flipping attacks and backdoor attacks. Under the label-flipping scenario, compromised clients maliciously invert the anomaly labels of their local training samples prior to optimization, aiming to confuse the global decision boundary and increase the false negative rate. In the backdoor attack scenario, malicious nodes inject targeted mathematical triggers into specific temporal windows of the sensing signals, forcing the model to systematically misclassify specific attack types as normal behaviors while maintaining high accuracy on clean data. The experiment compares the F1-scores of the standard FedAvg baseline and our proposed trust-aware method under attacker proportions of 20% and 30%, respectively, as shown in Table 7.

As observed in the extended evaluation results, the standard federated aggregation mechanism is highly vulnerable to these advanced poisoning strategies, exhibiting severe performance degradation, particularly when the attacker proportion reaches 30%. Standard averaging blindly incorporates the poisoned gradients, allowing the manipulated decision boundaries and hidden triggers to easily contaminate the global model. In contrast, the proposed trust-aware aggregation strategy successfully mitigates the destructive impact of both label-flipping and backdoor injections. By continuously evaluating the update-direction consistency and comparing parameter magnitude deviations against the trustworthy global median, the central server effectively isolates and down-weights the malicious updates. Consequently, our framework preserves the structural integrity of the global security representation space and ensures highly reliable anomaly detection even in severely compromised distributed environments.

### 4.7. System Overhead and Scalability Analysis

To comprehensively evaluate the operational feasibility and deployment potential of the proposed framework in real-world distributed security sensing systems, we conducted a systematic analysis of system overhead and scalability. The evaluation focuses on critical deployment metrics, including model parameter size, single inference latency, and the number of communication rounds required to achieve global convergence. The inference latency was measured locally on the client nodes to assess edge computational cost, while the communication cost was evaluated based on the transmission iterations of the shared encoder parameters. To further evaluate scalability, we varied the number of participating clients in the simulated network and analyzed the corresponding impact on detection performance and convergence stability under the established non-IID conditions.

As shown in Table 8, although the proposed method introduces a slight increase in model size and single inference latency due to the integration of the dual-attention mechanism and the personalized detection heads, the overall computational overhead remains highly acceptable for edge deployment. Crucially, the proposed method significantly reduces the number of communication rounds required for global convergence compared to standard federated baselines, which substantially lowers the communication bandwidth requirements in distributed networks. Furthermore, the scalability evaluation indicates that as the number of clients increases from the baseline configuration to fifty nodes, the framework maintains a highly stable F1-score with only minor fluctuations. This stable scalability is primarily attributed to the personalized federated optimization strategy, which effectively isolates local data heterogeneity within the customized detection heads and prevents large-scale client drift, demonstrating strong operational adaptability for large-scale distributed security sensing deployments.

### 4.8. Interpretability Analysis

**Figure 7:**
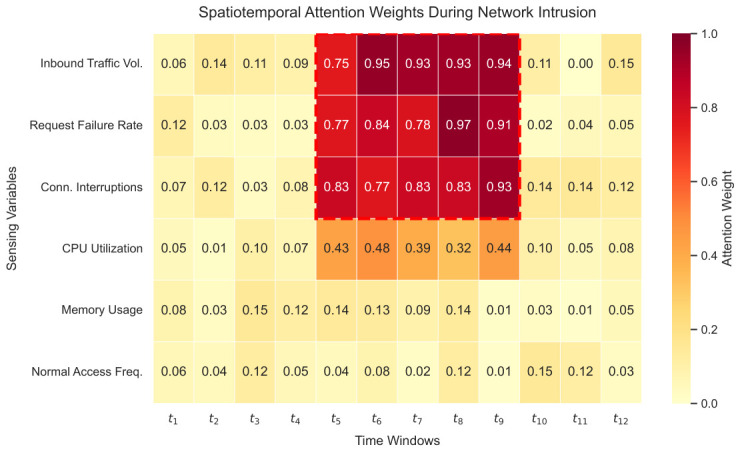
Visualization of spatiotemporal attention weights during a confirmed network intrusion event. The red dashed box highlights the critical time windows ($t_{5}$ to $t_{9}$) and the specific sensing variables (e.g., Inbound Traffic Volume, Request Failure Rate, and Connection Interruptions) that were most heavily weighted by the model’s dual-attention mechanism, demonstrating the interpretability of the diagnostic output.

Beyond detection accuracy, the interpretability of the proposed model plays a critical role in practical security monitoring environments. Recent work on explainable artificial intelligence has highlighted the paramount importance of transparent and interpretable pattern analysis [89]. Adapted to the cybersecurity context, this transparency is essential because security analysts must understand exactly why a system classifies a specific sequence as anomalous to facilitate rapid incident response, trace attack origins, and minimize false alarms. To demonstrate how the proposed temporal attention and feature attention mechanisms improve model explainability, we visualize the attention weight distributions during a confirmed anomaly event. As illustrated in Figure 7, the model generates a clear diagnostic output for a detected network intrusion attempt. The feature attention mechanism successfully assigns the highest impact weights to critical sensing variables, such as inbound traffic volume, request failure rate, and connection interruption counts, effectively isolating the exact feature dimensions manipulated by the attacker. Simultaneously, the temporal attention mechanism highlights the specific time windows where the anomalous burst initiated and peaked, assigning near-zero weights to the benign background traffic windows. This visual and tabular evidence confirms that the proposed framework does not operate as a black box; instead, it provides transparent, auditable, and operationally meaningful diagnostic insights, enabling security teams to rapidly pinpoint the root cause of an attack based on weighted sensing variables and critical time frames.

### 4.9. Discussion

The experimental results demonstrate that the proposed privacy-preserving federated deep anomaly detection framework has strong practical application value in distributed security sensing scenarios. Compared with traditional centralized anomaly detection methods, the proposed method is more suitable for deployment in real environments where multiple nodes, multiple devices, and multiple service systems coexist. For example, in large-scale campuses, data centers, industrial control sites, intelligent transportation hubs, or public service platforms, security data are usually distributed across servers, edge gateways, terminal devices, identity authentication systems, and network security devices. These nodes generate multisource data, such as service requests, access behaviors, network traffic, device temperature, load status, and alarm records, while also being constrained by permission boundaries, data compliance requirements, and service isolation policies. As a result, raw data are difficult to centralize directly on a unified platform for model training. In the proposed method, representation learning is completed at local nodes, and only the parameter updates of the shared encoder are uploaded. Therefore, different nodes can participate in collaborative modeling without exposing raw data, which better satisfies the deployment requirement of “data remaining within local domains while models remain collaborative” in practical security systems.

From the perspective of specific application scenarios, in data-center operation and maintenance security monitoring, different cabinets and server clusters may exhibit different variations in temperature, humidity, power consumption, fan speed, disk read/write rate, and network throughput. When abnormal heating, sudden increases in network connections, and disk I/O fluctuations occur in a group of servers, a single-node model may only identify local anomalies and may fail to exploit similar experiences from other nodes. Through the shared encoder, common risk patterns across server clusters can be learned, while local personalized detection heads can adapt to differences caused by different cabinets, service loads, and hardware configurations. In smart campuses or public service centers, access terminals, access control authentication devices, service interfaces, edge gateways, and security audit systems generate different types of security sensing signals. Anomalous behaviors may appear as short-term high-frequency access, remote login, abnormal interface invocation, terminal-state fluctuation, and correlated firewall alarms. Through the joint modeling of these multisource signals using temporal encoding and attention mechanisms, the proposed method can not only determine whether an anomaly exists, but also focus more effectively on key time windows and critical sensing variables, thereby improving the identification capability for complex anomaly chains.

Furthermore, the temporal modeling component of our framework demonstrates distinct advantages when positioned against existing sequence-based anomaly detection methods in dynamic environments. Recent studies have successfully employed recurrent architectures, such as hybrid LSTM models, to capture temporal dependencies in traffic-like security signals and dynamic anomaly patterns [90]. While such hybrid LSTM models are highly effective for sequential feature extraction in centralized or homogeneous settings, their hidden-state transmission mechanism can struggle to maintain stable global representations when deployed across non-IID federated clients. In contrast, the self-attention mechanism integrated into our proposed temporal encoder computes global correlations among temporal segments directly, without relying on sequential hidden-state updates. This non-sequential global dependency modeling ensures that long-range temporal associations are captured more robustly, which is particularly crucial when different clients exhibit varying sequence lengths, heterogeneous sampling frequencies, and highly skewed anomaly distributions. Consequently, our self-attention-based approach is inherently more suitable for the non-IID federated context, as it allows the shared encoder to aggregate invariant temporal patterns across distributed nodes without being biased by the local sequential variations that typically degrade the performance of conventional or hybrid recurrent networks.

The proposed method also has practical significance in terms of robustness. In scenarios such as industrial control, energy scheduling, and transportation infrastructure, attackers may not directly cause obvious failures. Instead, evasion detection may be attempted by slightly modifying access frequency, disguising normal requests, perturbing partial sensor readings, or creating local log missingness. Conventional models usually learn decision boundaries from clean samples and are prone to missed detections when facing such weak perturbation attacks. Through adversarial perturbation training and representation consistency constraints, relatively stable risk representations can still be maintained when input signals are affected by noise, masking, or camouflage. Therefore, the proposed method is more suitable for handling stealthy attacks in real security incidents. Meanwhile, during distributed collaborative training, some nodes may upload abnormal model updates because of device failures, data contamination, or attacks. The introduced trustworthiness evaluation and weighted aggregation mechanisms enable the server to reduce the influence of abnormal updates on the global model, preventing a single abnormal node from degrading the overall detection capability. Therefore, the proposed framework not only focuses on improving anomaly detection accuracy, but also emphasizes the unification of privacy protection, cross-node collaboration, local adaptation, and attack robustness, showing strong engineering adaptability for security monitoring systems involving multiple devices, multiple departments, and multiple edge nodes.

### 4.10. Limitation and Future Work

Several limitations remain in this study. First, the experimental scenarios mainly focus on structured temporal data in distributed security sensing systems, including terminal status, network communication, server operation, and anomaly alarms. Although such data can cover typical multi-node security monitoring requirements, the modeling of more complex multimodal data, such as images, textual logs, voice alarms, and device topology relationships, remains insufficient. Although the Transformer and attention mechanism architecture adopted in this framework possesses strong theoretical potential for handling multimedia feature embeddings due to its inherent sequence modeling and cross-modal alignment capabilities, extending it to process high-dimensional multimedia sensing data directly still faces substantial challenges. Specifically, extracting semantic features from video or audio streams requires significantly higher computational overhead, and aligning heterogeneous multimedia modalities within a unified non-IID federated environment demands more sophisticated attention designs. Second, the data-local federated learning mechanism adopted in this study mainly relies on the local retention of raw data. Although this strategy can reduce the leakage risk caused by centralized data aggregation, keeping data local does not fully establish privacy preservation. As highlighted in recent federated intrusion detection surveys [91], model updates may still leak sensitive information through gradient inversion, membership inference, or reconstruction attacks. Therefore, stronger security enhancements through formal privacy mechanisms, such as secure aggregation, differential privacy, secure multi-party computation, or encrypted communication, are necessary. Third, the trust-aware aggregation strategy can reduce the influence of abnormal client updates. However, when the proportion of malicious clients is high, when attackers perform coordinated camouflage, or when the distribution differences among normal clients are extremely large, trustworthiness estimation may still be disturbed.

Future work will be further extended in three directions. First, more types of security sensing data will be incorporated into a unified modeling framework, including unstructured log texts, device topology graphs, video surveillance information, and cross-system alarm links. We will further explore the extension of our attention-based temporal encoder to efficiently process multimedia feature embeddings, aiming to improve the comprehensive perception capability of the model for complex multimodal security events in edge intelligence contexts. Second, stricter formal privacy protection mechanisms, such as differential privacy and secure multi-party computation, will be introduced to enhance defense against parameter leakage and inference attacks while maintaining detection performance. Third, to address the extremely stringent requirements for low latency and real-time performance in actual engineering deployment, we will extensively explore the feasibility of deploying this framework in a lightweight manner on resource-constrained edge devices. Specifically, we intend to investigate knowledge distillation techniques to compress the complex global shared encoder into lightweight edge models, alongside model quantization to significantly reduce local computational overhead. Furthermore, we will focus on enhancing the capability of the framework for real-time online stream data processing by introducing streaming application programming interfaces and sliding window-based online optimization strategies. These approaches will enable the system to perform high-frequency, low-latency inference on continuous data streams, thereby effectively addressing the practical needs of the industrial sector and lending greater substance to our future research directions.

## 5. Conclusions

With the rapid expansion of distributed financial security sensing systems, achieving cross-node anomaly detection without centralizing raw transaction or client data, while handling non-IID transaction distributions and adversarial financial attacks, has become a critical challenge in financial cybersecurity. To address this issue, a data-local federated deep anomaly detection framework is proposed for financial scenarios, in which local representation learning, personalized federated optimization, and trust-aware aggregation are integrated into an end-to-end model. The main contributions of this study are threefold. First, a local representation module is constructed to extract anomaly-related features from core service transactions, payment interfaces, terminal access records, and system operational signals through temporal encoding and dual-attention mechanisms. Second, a personalized federated optimization mechanism is designed to separate a globally shared encoder from local detection heads, enabling cross-node knowledge sharing while adapting to the local data distributions of different financial nodes. Third, an adversarially robust and trust-aware aggregation strategy is introduced to enhance model stability under input perturbations, anomalous transaction interference, and malicious client updates. Extensive experiments demonstrate that the proposed method significantly outperforms standard federated baselines and robust federated baselines in financial security sensing tasks. In the main transaction anomaly detection task, an F1-score of 88.83% is achieved, and strong robustness is maintained under various adversarial financial conditions, including feature masking, noise interference, and malicious updates. Ablation studies further confirm the synergistic effectiveness of all integrated components. Despite these promising results, several limitations remain. The reliance on a hybrid financial dataset and simulated malicious transaction clients may not fully reflect the unpredictability of real-world financial cyberthreats. In addition, the current data-local paradigm still lacks rigorous mathematical privacy guarantees against advanced financial inference attacks, and the framework requires further validation in large-scale banking business and payment systems. Future research will focus on integrating formal privacy techniques such as differential privacy and secure multiparty computation, conducting multi-institutional empirical validation, enhancing explainable financial anomaly diagnosis, and exploring communication-efficient federated optimization for resource-constrained financial edge deployments.

## Figures and Tables

**Table 1:** Statistics of hardware sensing data in the distributed financial security sensing scenario.

Data Type	Data Quantity	Sensor Model
Front-end business terminal status data	420,000	Ingenico Lane/3000 Terminal Sensor
Self-service financial terminal operation data	310,000	NCR SelfServ Terminal Status Sensor
Edge gateway traffic data	840,000	Cisco ISR 4331 Flow Sensor
Switch port traffic data	530,000	Cisco Catalyst 9300 Port Sensor
Firewall traffic alarm data	126,000	Fortinet FortiGate 100F Security Sensor
Server hardware load data	720,000	Dell iDRAC 9 Hardware Sensor
Database server status data	360,000	Intel DCM Server Telemetry Sensor
Disk read/write status data	295,000	Seagate IronWolf SMART Sensor
Network interface throughput data	390,000	Intel X710 Network Adapter Sensor
Computer-room temperature and humidity data	285,000	Sensirion SHT31 Temperature-Humidity Sensor

**Table 2:** Non-independent and identically distributed data characteristics across federated financial clients.

Client ID	Node Type	Total Samples	Anomaly Ratio	Primary Anomaly Classes	Feature Characteristics
Client 1	Front-end business terminal	420,000	4.5%	Abnormal access, Short connection	Sporadic access patterns
Client 2	Self-service financial terminal	310,000	5.2%	Device restart, Interface error	Frequent state transitions
Client 3	Edge gateway	840,000	7.8%	Malicious IP access, DDoS	High inbound traffic
Client 4	Core switch	530,000	1.5%	Routing loop, ARP spoofing	High packet rate
Client 5	Firewall	126,000	18.4%	Rule violation, Malware signature	Concentrated alarm logs
Client 6	Core transaction server	720,000	3.6%	Traffic burst, System load anomaly	High CPU and memory variance
Client 7	Database server	360,000	2.1%	Disk I/O anomaly, Query timeout	Frequent disk read/write
Client 8	Storage server	295,000	1.8%	Read failure, SMART alarm	High I/O latency
Client 9	Identity access server	390,000	6.4%	Brute force, Token theft	High login failure counts
Client 10	Computer-room monitor	285,000	0.9%	Temperature burst, Power loss	Continuous environmental shift

**Table 3:** Comparison of anomaly detection performance under distributed security sensing scenarios.

Method	Accuracy (%) ↑	Precision (%) ↑	Recall (%) ↑	F1-Score (%) ↑	AUC (%) ↑	PR-AUC (%) ↑
Isolation Forest	82.41 ± 0.85	76.38 ± 0.92	71.52 ± 1.05	73.87 ± 0.96	80.36 ± 0.88	72.15 ± 1.12
Autoencoder	84.26 ± 0.76	78.44 ± 0.81	74.63 ± 0.89	76.49 ± 0.84	82.57 ± 0.75	74.91 ± 0.95
LSTM	86.15 ± 0.65	81.26 ± 0.72	78.34 ± 0.68	79.77 ± 0.69	85.28 ± 0.61	78.06 ± 0.78
Transformer	88.05 ± 0.58	83.74 ± 0.64	80.66 ± 0.61	82.17 ± 0.59	87.42 ± 0.54	80.71 ± 0.66
FedAvg	87.36 ± 0.72	82.58 ± 0.85	79.46 ± 0.78	80.99 ± 0.81	86.74 ± 0.68	79.88 ± 0.82
FedProx	88.24 ± 0.61	83.67 ± 0.73	80.92 ± 0.65	82.27 ± 0.67	87.96 ± 0.55	81.36 ± 0.71
SCAFFOLD	88.91 ± 0.54	84.38 ± 0.62	81.57 ± 0.59	82.95 ± 0.57	88.64 ± 0.49	82.11 ± 0.63
MOON	89.26 ± 0.48	85.12 ± 0.55	82.36 ± 0.52	83.72 ± 0.51	89.05 ± 0.43	82.97 ± 0.58
Ours	92.37 ± 0.35	89.41 ± 0.42	88.26 ± 0.38	88.83 ± 0.36	93.06 ± 0.31	89.15 ± 0.45

**Table 4:** Robustness comparison under input perturbations and malicious client attacks.

Method	Gaussian Noise	Feature Masking	Temporal Shift	Adv. Perturbation	20% Malicious	30% Malicious
FedAvg	76.84 ± 0.85	74.91 ± 0.92	75.36 ± 0.88	70.28 ± 1.12	72.43 ± 1.05	67.95 ± 1.25
FedProx	78.32 ± 0.78	76.45 ± 0.84	77.08 ± 0.81	72.16 ± 1.05	74.35 ± 0.95	70.24 ± 1.15
SCAFFOLD	79.15 ± 0.72	77.36 ± 0.79	78.22 ± 0.75	73.48 ± 0.98	75.91 ± 0.88	71.62 ± 1.08
FedNova	78.76 ± 0.75	76.94 ± 0.82	77.53 ± 0.78	72.85 ± 1.02	75.16 ± 0.92	70.83 ± 1.10
MOON	80.43 ± 0.65	78.82 ± 0.71	79.41 ± 0.68	74.96 ± 0.85	76.58 ± 0.82	72.47 ± 0.95
Median Agg.	81.16 ± 0.62	79.54 ± 0.68	80.02 ± 0.65	75.84 ± 0.82	79.68 ± 0.75	76.35 ± 0.88
Trimmed Mean	81.74 ± 0.58	80.21 ± 0.65	80.63 ± 0.61	76.51 ± 0.78	80.36 ± 0.72	77.42 ± 0.85
Krum	80.92 ± 0.61	79.13 ± 0.69	79.85 ± 0.64	76.02 ± 0.81	81.12 ± 0.70	78.26 ± 0.82
Ours	87.95 ± 0.45	86.42 ± 0.52	86.88 ± 0.48	84.57 ± 0.55	86.73 ± 0.42	83.91 ± 0.58

**Table 5:** Ablation study of the proposed privacy-preserving federated deep anomaly detection framework.

Variant	Accuracy (%) ↑	Precision (%) ↑	Recall (%) ↑	F1-Score (%) ↑	AUC (%) ↑	PR-AUC (%) ↑
w/o Local Deep Security Sensing Representation	88.13 ± 0.68	84.26 ± 0.75	81.74 ± 0.72	82.98 ± 0.70	88.41 ± 0.65	82.07 ± 0.78
w/o Temporal–Feature Attention Mechanism	89.02 ± 0.55	85.35 ± 0.62	82.91 ± 0.58	84.11 ± 0.60	89.23 ± 0.52	83.34 ± 0.64
w/o Personalized Aggregation Module	88.76 ± 0.61	84.91 ± 0.68	82.36 ± 0.64	83.61 ± 0.65	88.95 ± 0.57	82.86 ± 0.71
w/o Distribution Consistency Constraint	89.43 ± 0.52	85.86 ± 0.58	83.24 ± 0.55	84.53 ± 0.56	89.74 ± 0.49	83.95 ± 0.60
w/o Adversarial Training	89.68 ± 0.48	86.14 ± 0.54	83.71 ± 0.51	84.91 ± 0.52	90.08 ± 0.46	84.27 ± 0.56
w/o Trust-aware Aggregation Module	88.95 ± 0.59	85.02 ± 0.65	82.68 ± 0.61	83.83 ± 0.63	89.16 ± 0.54	83.02 ± 0.68
w/o Robust Components	87.84 ± 0.74	83.76 ± 0.81	81.39 ± 0.77	82.56 ± 0.78	88.02 ± 0.70	81.74 ± 0.84
Full Model	92.37 ± 0.35	89.41 ± 0.42	88.26 ± 0.38	88.83 ± 0.36	93.06 ± 0.31	89.15 ± 0.45

**Table 6:** Sensitivity analysis of trust-score regulatory coefficients under twenty percent malicious clients.

Parameter Variation	Evaluated Value	F1-Score (%)
Varying $\alpha_{1}$ (Direction Consistency)	0.1	79.35±0.62
	0.5	84.12±0.45
	1.0 (Default)	86.73±0.38
	2.0	86.51±0.41
Varying $\alpha_{2}$ (Magnitude Deviation)	0.1	81.24±0.55
	0.5	85.66±0.42
	1.0 (Default)	86.73±0.38
	2.0	83.45±0.58
Varying $\alpha_{3}$ (Historical Reliability)	0.1	83.18±0.47
	0.5 (Default)	86.73±0.38
	1.0	86.42±0.44
	2.0	85.19±0.52

**Table 7:** Robustness comparison under label-flipping and backdoor attacks.

Attack Scenario	Attacker Proportion	FedAvg F1-Score (%)	Proposed Method F1-Score (%)
Label-flipping	20%	68.45±0.85	85.34±0.46
	30%	59.12±1.05	82.71±0.53
Backdoor Attack	20%	71.28±0.76	84.92±0.49
	30%	63.55±0.94	81.65±0.61

**Table 8:** System overhead and operational metric comparison.

Method	Model Size (MB)	Inference Latency (ms)	Communication Rounds
FedAvg	18.4	5.2	85
FedProx	18.4	5.2	82
Proposed Method	21.6	6.7	64

## Data Availability

The data presented in this study are available on request from the corresponding author due to privacy and confidentiality restrictions associated with the original research data, including non-public project data and information related to the sampling sites.
